# Not all β-lactams are equal regarding neurotoxicity

**DOI:** 10.1186/s13054-016-1522-z

**Published:** 2016-10-27

**Authors:** Khalil Chaïbi, Maïté Chaussard, Sabri Soussi, Matthieu Lafaurie, Matthieu Legrand

**Affiliations:** 1 Department of Anesthesiology and Critical Care and Burn Unit, St-Louis Hospital, Assistance Publique- Hopitaux de Paris, Paris, France; 2 University Paris Diderot, Paris, France; 3 UMR INSERM 942, Institut National de la Santé et de la Recherche Médicale (INSERM), Lariboisière Hospital University Paris Diderot, F-75475 Paris, France; 4 AP-HP, Infectious disease, St-Louis Hospital, Paris, France

We read with interest the letter from May et al. recently published in *Critical Care* [1]. The authors suggest that overdosing of β-lactams in 108 critically ill patients receiving renal replacement therapy may not be associated with neurotoxicity. We agree that adequate plasmatic levels of β-lactams in critically ill patients with sepsis should be a primary goal and that under-dosing may put the patient at a dangerous risk of treatment failure [2]. We are, however, concerned about the conclusions of this letter and would like to comment on two points.

First, not all β-lactams are equal regarding neurotoxicity, and some may expose the patient to higher risk. A number of studies have reported neurotoxicity of imipenem, especially in patients with brain injury [3, 4]. More recently, several authors have reported neurotoxicity with cefepime. Fugate et al. reported that 7 % to 15 % of critically ill patients treated with cefepime developed definitive or possible neurotoxicity [5]. Hence, renal insufficiency was a major risk factor. We have also observed over the last 2 years similar findings with four cases of cefepime-related neurotoxicity in our intensive care unit (ICU) in patients with chronic renal insufficiency or acute kidney injury.

Second, May et al. defined neurotoxicity as an association of overdose and convulsions. There is, however, strong evidence showing that neurotoxicity of β-lactams can present without seizures [6]. Cefepime-related neurotoxicity has been reported to present mostly as myoclonus and impaired consciousness, confusion, hallucinations, or agitation. Convulsions might be only the most extreme manifestation of the induced encephalopathy toxicity. In our cases, the neurological manifestations were loss of contact, agitation, disorientation and, for two of them, myoclonic twitches and jerks. The observed cefepime trough level of those patients during these events ranged from 144 mg/l to 455 mg/l for an upper therapeutic trough level of 90 mg/l according to our laboratory, while daily doses were 4 to 6 g/day. An electroencephalogram was carried out for each patient and has objectified a toxic encephalopathy pattern or aspecific slow waves with no sign of lobe epilepsy. Every patient experienced a regression of the symptoms with the withdrawal of the offending drug.

To conclude, while we agree that high serum levels of β-lactams should be obtained at the initiation of antibiotic therapy in patients with sepsis, intensivists should not overlook the potential neurotoxicity of some β-lactams with a low therapeutic index such as imipenem and cefepime. The risk of cefepime neurotoxicity should not be underappreciated in patients with neurological symptoms, especially if renal function is impaired.

## Abbreviations

BL: Beta-lactams
ICU: Intensive care unit
ARF: Acute renal failure
NCSE: Non-convulsive status epilepticus
