# Supplementary material for: Not all β-lactams are equal regarding neurotoxicity
Source: Crit Care. 2016 Oct 27;20:350. doi: 10.1186/s13054-016-1522-z (PMC5081676; doi:10.1186/s13054-016-1522-z)
Supplement: Related texts

# Reply: Authors’ response

**Authors:** Faten May, Najouah El-Helali, Jean-François Timsit, Benoît Misset

We thank Dr Chaïbi et al. for their valuable and insightful comments on our report of the low evidence for an association between supra-therapeutic serum levels of β-lactams (BL) and clinical toxicity in ICU patients with acute renal failure (ARF) treated with intermittent hemodialysis [1].

Chaïbi et al. point out that neurotoxicity is common among BL, especially in at-risk patients such as those with ARF, and that some BL with a low therapeutic index may induce more neurological events. Several reports described an increased risk for penicillins, imipenem, and fourth generation cephalosporins, and suggested that neurological complications may range from confusion, depressed level of consciousness, and non-specific encephalopathy to myoclonus, non-convulsive status epilepticus (NCSE), and seizures [6].

We would like to draw attention to the fact that the majority of those articles were non-human experimental studies, case series, and retrospective reviews, with assessment offering low levels of evidence [7]. To establish a cause-effect relationship between a BL administration and clinical signs of encephalopathy remains a major difficulty because both clinical and electrical signs are non-specific. All the neurological symptoms of clinical encephalopathy may be observed in conditions such as metabolic abnormality, uremic disorder, ICU delirium, withdrawal syndrome, or septic encephalopathy, which are very frequent in ICU septic patients [8].

In our population, while we may have missed non-convulsive signs and NCSE, we could not find an association between convulsions and BL observed at the supra-therapeutic level.

Therefore, despite the comments of Chaïbi et al., we consider that the BL neurotoxic threshold is not yet well identified and is still challenging. A proper diagnosis of BL neurological toxicity may be obscured by the overall clinical picture and may easily be attributed to the infectious process or to an underlying metabolic. We estimate that prospective observational studies, including pre-established and precise definitions and design, are still needed to assess BL encephalopathy and the neurotoxic threshold and to conclude on their clinical impact.
